# Retrospect of the Medical Sciences

**Published:** 1843-12-09

**Authors:** 


### DR. TAVERNIER'S LEVER-BELT AND BUSK FOR THE TREATMENT OF SPINAL DEFORMITIES.

The apparatus consists of a broad leather belt, which fits round the pelvis, and has a kind of semicircular steel rack attached to it at a point corresponding to the sacrum. A steel bar or busk, which fits vertically into this rack, can be placed and firmly fixed in any requisite degree of lateral inclination. A broad leather strap is fastened to the belt in front, and is destined, after being brought obliquely round the body, to be fastened by two or more tongues to studs towards the upper part of the busk. The belt round the pelvis is steadied by a strap passed underneath the thigh, in order to render it a sufficiently fixed point of resistance.

To apply this instrument, suppose in a case of right lateral curvature, the belt is first fastened rather loosely round the pelvis, and the busk is inclined laterally towards the left shoulder, the degree of its inclination depending on the amount of the deformity. The strap, which is fastened to the belt in front, is now passed obliquely in front of the thorax and under the right arm, so as to pass over the ribs, which project in consequence of the spinal deformity. The length of this strap is so adjusted that it cannot reach the busk while the body is erect, and the trunk must therefore be bent to the left side, and while it is in that position the strap is fastened to the busk. The patient is now obliged to endeavour to throw the body to the right side, in order to restore the equilibrium; and, to use Dr. Tavernier's own words, "by this movement, which that part of the spine situated above the strap can alone perform, the upper half of the arch, which the curved spine presents, finds itself of necessity brought back, in conformity to the axis of the body."

We may quote the following explanation of the mode of its action:

"It is then, as we may see, by the combination of two actions, one mechanical, and produced by the apparatus, the other physiological, resulting from the play of the muscles, that the method of the lever with inclination acts on the bent spine. It is, in other words, working to obtain an opposite effect to that which we desire to destroy, and by the intervention of the same forces, the same immediate causes, but acting in a contrary direction, and with the view of effecting a cure, that this apparatus acts beneficially on the vertebral column."

The reviewer in the British and Foreign Medical Review, for October, considers it as really a very valuable instrument in the treatment of lateral curvature of the spine. The reviewer has applied it in two cases, and in both was the result most satisfactory.

In order to render the cure permanent, Dr. Tavernier recommends the use of "a simple ordinary corset, to which are differently adapted, according to the case, large ribands, which have for their object to bend the spine in the direction of the busk employed for the treatment."

---

### DR. BENNETT ON INFLAMMATION OF THE NERVOUS CENTRES.

1. Two kinds of cerebral and spinal softening exist, an inflammatory and a non-inflammatory, which may always be distinguished from each other by means of the microscope.

2. Inflammatory softening is characterized by the presence of exudation corpuscles and granules, whilst in non-inflammatory softening these bodies are never found.

3. The nature of inflammatory softening consists in the formation and development of nucleated cells in exuded blood plasma; whilst the nature of non-inflammatory softening consists in the mechanical destruction or maceration of the nervous tissue in serum, or is the result of putrefaction.

4. Non-inflammatory softening, unaccompanied by hemorrhage, is usually *post mortem*, and causes no symptoms; whilst uncomplicated inflammatory softening always causes marked symptoms, which, however, vary according to the seat of the lesion.

5. The inflammatory and non-inflammatory softenings have frequently been confounded together by morbid anatomists, it being impossible to distinguish one from the other, with any certainty, by the naked eye.

6. Inflammation in the nervous centres has, in several instances, been demonstrated by means of the microscope, after it had escaped the search of good morbid anatomists, and been indicated by the most unequivocal symptoms.

7. Every different coloured softening has, at various times, been found to be connected with inflammation, but that yellow and white softenings are most frequently non-inflammatory, whilst the fawn-coloured softening is commonly inflammatory.

8. Red softenings usually depend on congestion, or the direct extravasation of blood; yellow softenings, on the imbibition of the colouring matter of the blood; fawn and gray-coloured softenings on the presence of brown exudation corpuscles; and white softenings, in the great majority of cases, are *post mortem* and the result of maceration in serum.

9. In no single instance has softening of the nervous centres been traced to the presence or infiltration or pus.

10. Inflammation of the central parts of the brain generally produce well-marked lesions of sensation and motion, whilst, in inflammation of the peripheral portions, lesions of intelligence are commonly well pronounced.

11. In idiopathic inflammatory softening of the brain, contraction in one or more limbs is a common symptom.

12. The fawn-coloured spots described by Dr. Sims are no evidence of the cure of inflammatory softening.

13. Inflammation accompanying hemorrhages is usually consecutive.

14. The softening surrounding apoplectic clots or sanguineous infiltration is no proof of inflammatory action.—*Edin. Med. and Sur. Journal,* October 1, 1843.

